# Mig6 Is a Sensor of EGF Receptor Inactivation that Directly Activates c-Abl to Induce Apoptosis during Epithelial Homeostasis

**DOI:** 10.1016/j.devcel.2012.08.001

**Published:** 2012-09-11

**Authors:** Sarah Hopkins, Emma Linderoth, Oliver Hantschel, Paula Suarez-Henriques, Giulia Pilia, Howard Kendrick, Matthew J. Smalley, Giulio Superti-Furga, Ingvar Ferby

**Affiliations:** 1 Wolfson Institute for Biomedical Research, University College London, Gower Street, London WC1E 6BT, UK; 2 CeMM – Center for Molecular Medicine of the Austrian Academy of Sciences, Lazarettgasse 14, 1090 Vienna, Austria; 3 Ludwig Institute for Cancer Research, Science for Life Laboratory, Uppsala University, 751 24 Uppsala, Sweden; 4 Breakthrough Breast Cancer Research Centre, Institute of Cancer Research, 237 Fulham Road, London SW3 6JB, UK

## Abstract

A fundamental aspect of epithelial homeostasis is the dependence on specific growth factors for cell survival, yet the underlying mechanisms remain obscure. We found an “inverse” mode of receptor tyrosine kinase signaling that directly links ErbB receptor inactivation to the induction of apoptosis. Upon ligand deprivation Mig6 dissociates from the ErbB receptor and binds to and activates the tyrosine kinase c-Abl to trigger p73-dependent apoptosis in mammary epithelial cells. Deletion of *Errfi1* (encoding Mig6) and inhibition or RNAi silencing of c-Abl causes impaired apoptosis and luminal filling of mammary ducts. Mig6 activates c-Abl by binding to the kinase domain, which is prevented in the presence of epidermal growth factor (EGF) by Src family kinase-mediated phosphorylation on c-Abl-Tyr488. These results reveal a receptor-proximal switch mechanism by which Mig6 actively senses EGF deprivation to directly activate proapoptotic c-Abl. Our findings challenge the common belief that deprivation of growth factors induces apoptosis passively by lack of mitogenic signaling.

## Graphical abstract

**Graphical Abstract:**

**Figure:**
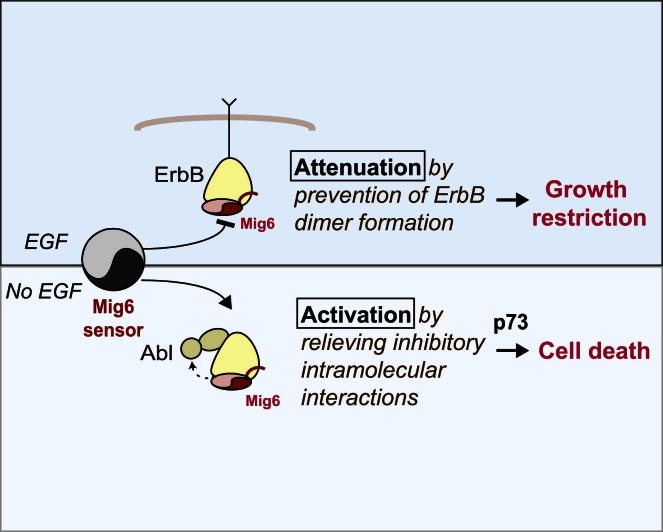

**Highlights:**

► EGFR inactivation triggers “inverse signaling” by Mig6-mediated activation of Abl ► Mig6 is a bimodal switch that attenuates EGFR (+EGF) or activates c-Abl (-EGF) ► Mig6 activation of Abl regulates cell death during mammary epithelial homeostasis

## Teaser

Hopkins et al. show that tyrosine kinase signaling directly links EGF receptor (ErbB) inactivation to the induction of apoptosis. Inactivation dissociates ErbBs from the Mig6 tumor suppressor, which then binds and activates c-Abl to trigger p73-dependent apoptosis. This mechanism controls mammary epithelial homeostasis and may also contribute to oncogene addiction.

## Introduction

Most epithelial cell types rely on signaling controlled by members of the four epidermal growth factor (EGF) receptor-tyrosine kinases, EGFR and ErbB2-4, to regulate cellular functions as diverse as proliferation, survival, cell differentiation, and motility (Avraham and Yarden, 2011; Linggi and Carpenter, 2006). Conversely, their deregulation disrupts normal tissue homeostasis and contributes to the formation of a vast proportion of epithelial cancers. Ligand binding causes ErbB receptor homo- or heterodimerization, which leads to allosteric induction of their intrinsic tyrosine kinase activity and subsequent auto- or transphosphorylation (Jura et al., 2009; Red Brewer et al., 2009; Scheving et al., 2006). Furthermore, compelling evidence indicate that the ErbB receptors synergize with the membrane bound nonreceptor tyrosine kinase Src for full receptor activation. Src associates with the ErbB receptors via its kinase domain to further phosphorylate them on multiple important tyrosine residues (Tice et al., 1999; Kim et al., 2005; Sato et al., 1995).

The ErbB receptors are under tight spatiotemporal control, in part through negative feedback loops to ensure correct signaling outcome (Avraham and Yarden, 2011). The multiadaptor protein Mig6, also called RALT, is a negative feedback regulator of the ErbB receptors that acts by directly binding to the active receptor kinase domain thereby interfering with the formation of the activating dimer interface (Zhang et al., 2007a). In addition Mig6 plays a role in endosomal targeting of the receptors for degradation (Fiorentino et al., 2000; Frosi et al., 2010; Jura et al., 2009; Ying et al., 2010; Zhang et al., 2007a). The physiological importance of Mig6 is evident by recent findings that *Errfi1* (encoding Mig6) null mice exhibit sustained ErbB signaling and develop hyperplasia and tumors in various tissues (Ferby et al., 2006; Zhang et al., 2007b). Furthermore, Mig6 is frequently downregulated in various types of human cancers, consistent with an important tumor suppressive function for Mig6 (Amatschek et al., 2004; Ferby et al., 2006; Reschke et al., 2010).

Individual cells express numerous members of the receptor tyrosine kinases superfamily many of which activate the same canonical mitogenic signaling pathways, yet cells depend on specific growth factors for their survival. Here, we identify a Mig6-controlled receptor-proximal switch mechanism that directly links ErbB receptor inactivation to the activation of the proapoptotic c-Abl, thereby establishing cellular dependence on EGF in mammary epithelial cells. Interfering with Mig6 function by gene targeting or loss of c-Abl function leads to disrupted mammary morphogenesis characterized by ductal luminal filling due to impaired cell death.

## Results

### Loss of Mig6 Leads to Impaired Epithelial Cell Death during Mammary Ductal Morphogenesis

**Figure 1:**
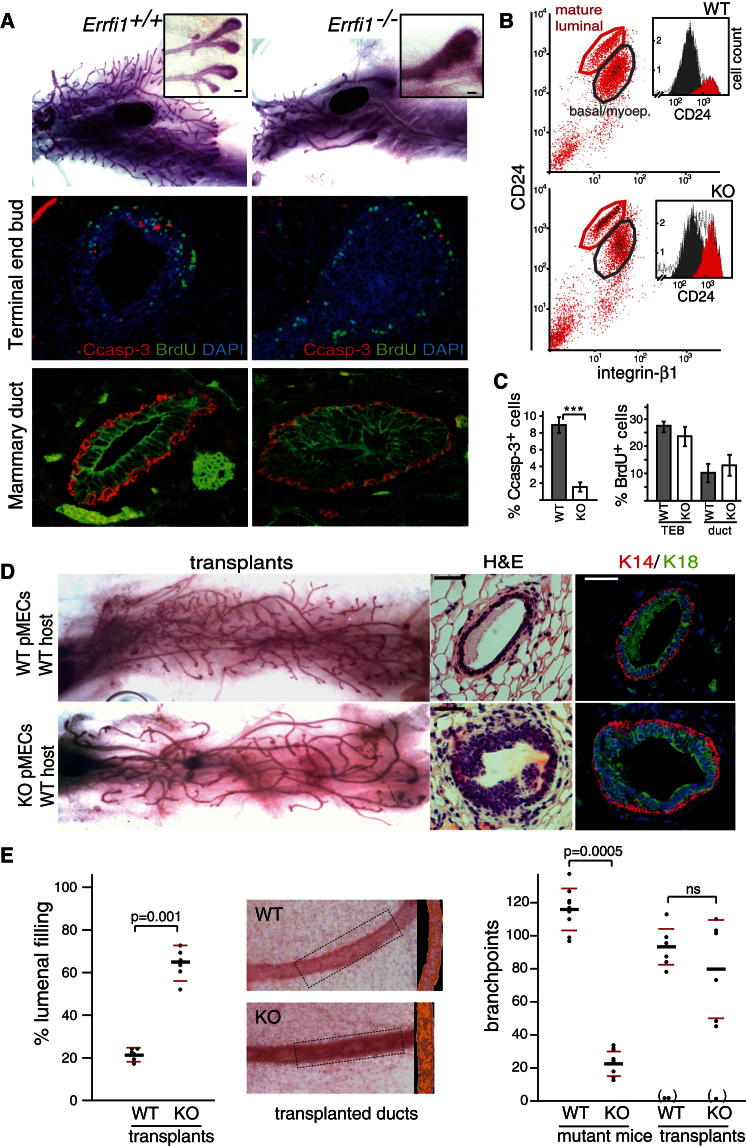
Deletion of *Errfi1* Leads to Impaired Cell Death and Propagation of Luminal Epithelial Cells during Mammary Morphogenesis (A) Mammary glands from BrdU-injected 6-week-old *Errfi1*^*−/−*^ or wild-type littermate control mice were either subjected to whole-mount carmine staining (upper panels) or terminal end buds and mammary ducts were subjected to immunostaining for cleaved caspase 3 (Ccasp-3), or BrdU as indicated. Mammary ducts from a 10-week-old *Errfi1*^*−/−*^ or wild-type littermate control mice were stained for keratin-14 (K14) or keratin-18 (K18) as indicated. (B) pMECs were isolated from 14- to 15-week-old *Errfi1*^*−/−*^ (KO) or wild-type (WT) littermate mice and subjected to flow cytometry with antibodies against indicated proteins. The number of CD24^high^ mature luminal cells (circled red) and CD24^low^ basal/myoepithelial cells (circled gray) are represented in the inlayed graphs. (C) Quantification of cleaved caspase 3^+^ cells in terminal end buds of KO or WT littermate mammary glands, and % BrdU^+^ cells in TEBs and ducts analyzing longitudinal sections from mice aged 6–8 weeks. Data were analyzed by two-tailed unpaired Student's t test (p = 0.0023, denoted three asterisks), while error bars represent SEM. n = 14 (Ccasp-3) and n = 12 (BrdU) independent TEBs from five mice (WT), and n = 9 (Ccasp-3) and n = 9 (BrdU) TEBs from five mice (KO); and for ductal BrdU incorporation: n = 15 (WT) and n = 12 (KO) ducts from six and five mice, respectively. (D) Epithelial cell autonomous function of Mig6. Cleared mammary fat pad transplantation of pMECs isolated from WT or KO littermates into WT hosts and analysis of mammary outgrowths by carmine staining or mammary ducts by H&E or immunostaining for K14 and K18 as indicated. (E) The degree of filling of primary ducts was quantified as the ratio of higher intensity areas (orange) of carmine staining over the total ductal area (middle panels show representative sections). Dots in the left hand graph represent the mean high intensity area/total ductal area per mouse. The number of branchpoints per gland was determined comparing glands from *Errfi1*^*−/−*^ (KO) mice or littermate wild-type (WT) controls plotted individually (n = 9 for WT and KO mice; n = 6 for WT and KO transplants. Failed transplant outgrowths were excluded (in parenthesis). Data were analyzed by two-tailed unpaired Student's t test. The black bar indicates the mean while red bars represent SEM. Scale bars: 1 mm (whole mounts) and 25 μm (H&E and immunofluorescence images). See also Figure S1.

To explore the mechanism by which Mig6 regulates epithelial morphogenesis, we examined the development of the mammary gland in the *Errfi1* null mice. We found that loss of Mig6 leads to disruption of ductal morphogenesis of the mammary gland characterized by filling of terminal end buds and mammary ducts and a 5-fold decrease in ductal branching (Figures 1A and 1E; Figure S1A available online). Histological analysis of cross sections of the mammary ducts from pubertal and adult mammary glands revealed a multilayered, epithelium (Figure 1A; Figure S1A) with widespread luminal filling (Figure 1E) in the *Errfi1*^*−/−*^ mice. The expanded cell layers constitute luminal epithelial cells rather than basal/myoepithelial cells, as shown by immunostaining for the differentiation basal/myoepithelial marker keratin-14, and markers for luminal cells: keratin-18 (Figure 1A), E-cadherin, and GATA-3 (Figure S1A). To confirm the selective propagation of luminal over basal/myoepithelial cells, we performed flow cytometrical analysis of freshly isolated *Errfi1*^*−/−*^ and wild-type mammary cells. The *Errfi1*^*−/−*^ primary mammary epithelial cells (pMECs) showed a 3-fold increase in the CD24^high^/integrin-β1^low^ mature luminal cell population, relative to the CD24^low^/integrin-β1^high^ basal/myoepithelial cell population (Figure 1B).

Bim (Bcl-2 interacting mediator of cell death)-dependent apoptosis has previously been shown to play a crucial role in the process of lumen formation of the mammary gland (Mailleux et al., 2007), suggesting that the luminal filling in the *Errfi1*^*−/−*^ mice may be due to impaired cell death. In agreement, invading terminal end buds (TEBs) of pubertal *Errfi1*^*−/−*^ mammary glands showed a 7-fold decrease in cell death as compared to wild-type littermates, as determined by quantification of cleaved caspase-3 positive cells (Figure 1C). In contrast, cell proliferation (incorporation of bromodeoxyuridine [BrdU]) was not affected neither in TEBs nor ducts by loss of *Errfi1* (Figures 1A and 1C). In addition, *Errfi1*^*−/−*^ pMECs proliferated at the same rate as wild-type control cells in vitro (Figure S1B). Together, these results show that loss of Mig6 leads to selective propagation of luminal epithelial cells due to impaired cell death rather than increased cell proliferation.

### Mig6 Is Required Cell Autonomously in Epithelia for Luminal Clearance

Ductal morphogenesis of the mammary glands involves both epithelial cell autonomous, micro-environmental and paracrine factors. Notably, Mig6 is also expressed in fibroblasts surrounding the mammary ducts (data not shown). To assess the epithelial-specific requirement for Mig6 in mammary morphogenesis, we transplanted pMECs isolated from *Errfi1*^*−/−*^ or wild-type littermate mice into cleared fat pads of wild-type recipient mice and characterized reconstituted glands by whole-mount carmine staining and IHC on ductal cross sections (Figure 1D). *Errfi1*^*−/−*^ glands in a wild-type background recapitulated the ductal luminal filling phenotype, as quantified by optical density analysis of carmine stained primary ducts (Figure 1E), demonstrating that Mig6 regulates luminal clearance in a cell autonomous fashion in the epithelia during mammary morphogenesis. Determination of the number of branchpoints in the full *Errfi1* null mice as compared with wild-type controls revealed a 5.5-fold decrease in ductal branching, while there was greater variability and no statistically significant difference in branching between wild-type and *Errfi1* knockout glands reconstituted in wild-type hosts (Figure 1E). These results indicate a possible involvement of Mig6 in the microenvironment for branching morphogenesis, while it has a cell-autonomous function in epithelia to mediate luminal clearance. Further studies will be required to address the role of Mig6 in branching morphogenesis

To explore the cellular mechanism by which Mig6 regulates mammary epithelial cell death, we first characterized *Errfi1*^*−/−*^ pMECs cultured in vitro. These cells exhibit normal cell proliferation rate as shown by the MTT reduction assay (Figure S1B). In addition, *Errfi1*^*−/−*^ pMECs shown normal cellular morphology as indicated by phase contrast microscopy (Figure S2A). Epithelial cell differentiation also appeared unaffected by loss of Mig6 as shown by immunoblotting against differentiation markers keratin-14, -18, E-Cadherin, β-catenin, Musashi, and GATA-3 (Figure S2B).

**Figure 2:**
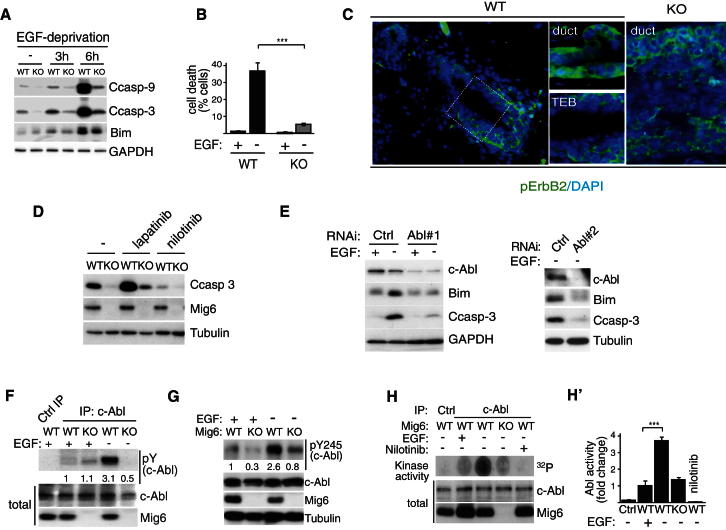
c-Abl Is Activated upon EGF Deprivation in a Mig6-Dependent Manner and Required for Apoptosis (A) *Errfi1*^*−/−*^ or wild-type pMECs were cultured in the presence (+) or absence (−) of 10 ng/ml EGF for indicated times and cell lysates subjected to immunoblotting as indicated. (B) Trypan blue cell viability assay on *Errfi1*^*−/−*^ or wild-type pMECs cultured in the presence (+) or absence (−) of 10 ng/ml EGF for 8 hr. The % of cells taking up the dye is indicated (n = 3 independent experiments; ^∗∗∗^p = 0.00015). (C) Longitudinal sections of a TEB or duct from wild-type (WT) or *Errfi1*^*−/−*^ (KO) mice (5 and 6 weeks of age, respectively) subjected to immunostaining for active phospho-Y877-ErbB2 and counterstained with DAPI. Note the absence of active ErbB2 in the interior luminal cell layers in the wild-type TEB or *Errfi1*^*−/−*^ duct. Representative images of eight analyzed TEBs and ducts from a 6-week-old mouse. (D) *Errfi1*^*−/−*^ (KO) or littermate wild-type pMECs (WT) were deprived of EGF for 6 hr in the absence (−) or presence (+) of 1 μM lapatinib, 1 μM nilotinib, prior to western analysis for indicated proteins. The right panels constitute a separate independent experiment. Note that inhibition of c-Abl disrupts EGF-deprivation induced cell death, while inhibition of EGFR and ErbB2 fails to rescue the cell death defect of the KO pMECs. (E) pMECs subjected to shRNAi-mediated knock-down of c-Abl using two independent constructs were deprived of EGF for 6 hr and cell lysates immunoblotted for indicated proteins. (F and G) pMECs from *Errfi1*^*−/−*^ or littermate wild-type mice were cultured in the presence or absence of EGF for 2 hr prior to harvest, followed by immunoprecipitation of c-Abl and immunoblotting for total phospho-tyrosine (F) or tyrosine 245 (G, independent experiment), indicating that c-Abl is rapidly activated upon EGF withdrawal in a Mig6-dependent manner. The numbers indicate fold increase in intensity as determined by densitometric analysis. (H and H′) pMECs from KO or littermate WT mice were cultured in the presence or absence of EGF for 2 hr prior to harvest, followed by immunoprecipitation of c-Abl and an in vitro kinase assay using a specific Abl substrate peptide (Abltide) as a substrate. (H′) Quantification of kinase activity as fold change over the EGF stimulated WT samples (n = 4, ^∗∗∗^p = 0.00017). See also Figures S2 and S3). Indicated p values were determined by two-tailed unpaired Student's t test, while the error bars represent SEM.

pMECs require an EGF ligand for survival and removal of EGF from otherwise growth factor supplemented media triggers rapid cell death in wild-type pMECs (Figure 2A). *Errfi1*^*−/−*^ cells, however, were resistant to cell death induced by EGFR inactivation, as determined by cleavage of caspases-3 and -9, induction of Bim (Figure 2A) or cell membrane permeability (Figure 2B). We asked whether Mig6 has a more general role in promoting cell death. This was not the case since neither anoikis nor DNA damage (adriamycin)-induced cell death was affected by loss of Mig6 (Figures S2C and S2D).

We then addressed whether luminal cells are subjected to inactivation of ErbB signaling in vivo by examining cross sections of TEBs for immunoreactivity against active ErbB2 using a phosphospecific antibody. While active ErbB2 was readily detectable in the basal cell layers of the TEB as well as ductal luminal cells, cells in the interior of TEBs show only limited or undetectable levels of phospho-ErbB2 staining (Figure 2C). Inactivation of ErbB receptors may therefore contribute to cell death and luminal clearance of TEBs.

### Mig6 Regulates Mammary Epithelial Cell Death Independent of Its Role as a Negative Regulator of ErbB Signaling

Given the known function of Mig6 as a negative regulator of the ErbB receptors (Ferby et al., 2006; Frosi et al., 2010; Zhang et al., 2007b) we asked whether gain of basal ErbB kinase activity underlies the acquired EGF-independent survival of *Errfi1* mutant cells. However, western analysis of *Errfi1*^−/−^ and wild-type pMECs did not show an increase in EGFR activation or mitogenic signaling via Erk1/2, Akt, and GAB2 neither in the presence nor absence of EGF (Figures S2B and S2E). Furthermore, treatment of pMECs with the specific ErbB inhibitors AG825 (ErbB2), gefitinib (EGFR), or lapatinib (EGFR and ErbB2) prior to EGF deprivation failed to rescue the EGF-independent survival of *Errfi1*^*−/−*^ cells (Figures 2D and S2F). These results suggest that Mig6 regulates mammary epithelial cell death independent of its role as a negative regulator of the ErbB receptors.

### EGFR Inactivation Leads to Mig6-Dependent Activation of Abl and Induction of Cell Death

In order to identify the putative survival or apoptotic pathway regulated by Mig6 we used specific inhibitors against kinases known to play a role in survival signaling downstream of the ErbB receptors including MEK, PI-3K, the Src family of kinases, c-Met, and c-Abl and analyzed their effect on apoptosis triggered by EGF-deprivation in wild-type and *Errfi1*^*−/−*^ pMECs (Figures S2F and S2G). While all inhibitors failed to rescue the Mig6-dependent apoptotic response, treatment with the specific second generation c-Abl inhibitor nilotinib (Tasigna) (Hantschel et al., 2008), prevented cell death in response to EGFR inactivation (Figure 2D). To confirm whether c-Abl is the relevant target of nilotinib, we performed RNAi-mediated knockdown of c-Abl in pMECs using two nonoverlapping shRNAs that similarly reduced cell death induced by EGF deprivation (Figures 2E). These results show that c-Abl is required for cell death induced by EGF deprivation.

Substantial evidence from cultured cells support a role for c-Abl in promoting cell death in response to different cellular stresses, including DNA damage, reactive oxygen species, ER stress or by the proapototic cytokine TNFα (Agami et al., 1999; Chau et al., 2004; Gong et al., 1999; Ito et al., 2001; Noren et al., 2006; Sun et al., 2000), although in vivo evidence remains limited primarily due to neonatal lethality of Abl knockout mice (Tybulewicz et al., 1991). To investigate whether c-Abl is activated upon EGF deprivation, wild-type and *Errfi1*^*−/−*^ pMECs were cultured with or without EGF, followed by examination of c-Abl phosphorylation on either all tyrosine residues (pY) or the regulatory Y245 (pY245) alone by western analysis of immunoprecipitated Abl (Figures 2F and 2G). Strikingly, tyrosine phosphorylation of c-Abl was readily detectable already after 2 hr following EGF withdrawal prior to caspase-3 cleavage (Figures 2F and 2G) and remained phosphorylated for up to 12 hr (data not shown). In agreement, immunoprecipitated c-Abl showed a 3.6-fold increase in kinase activity upon EGF withdrawal (Figures 2H and 2H′). On the contrary, phosphorylation and activation of c-Abl were not induced in *Errfi1*^*−/−*^ pMECs. Taken together, these results show that Mig6-dependent activation of c-Abl is required for mammary epithelial cell death in response to EGF deprivation.

### Mig6 Binds to and Activates c-Abl

**Figure 3:**
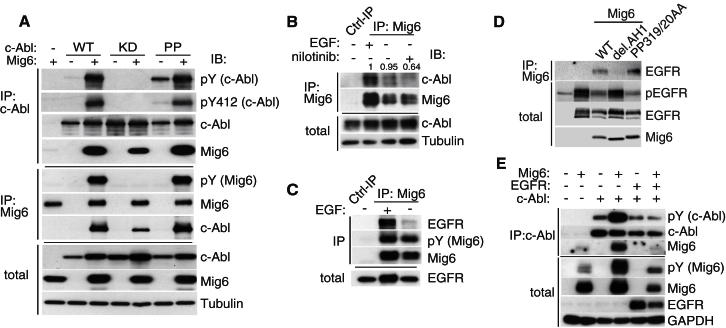
Mig6 Interacts with and Activates c-Abl (A) c-Abl or Mig6 were immunopurified from HEK293T cells ectopically coexpressing Mig6 and wild-type (WT), catalytically inactive (KD), or constitutively active (PP) forms of c-Abl and examined by immunoblotting as indicated. (B and C) Immunopurification of endogenous Mig6 from pMECs cultured in the presence or absence of EGF for 2 hr and examined for interaction with c-Abl (B) or EGFR (C). (D) Immunoprecipitated EGFR from HEK293T cells ectopically coexpressing EGFR and Mig6 or mutant forms of Mig6 lacking the AH1 domain (deletion aa 328–361) or the Grb2 binding site (PP319/20AA) and examined by immunoblotting as indicated. (E) Immunoprecipitation of c-Abl coexpressed with Mig6 and EGFR in HEK293T cells followed by immunoblotting as indicated.

To explore the mechanism by which Mig6 contributes to c-Abl activation, we first addressed whether the two proteins physically interact. Indeed, ectopically expressed Mig6 coimmunoprecipitated with wild-type (WT), kinase-dead (KD), and constitutively active (PP) forms of c-Abl (Figure 3A). Strikingly, Mig6 triggered activation of c-Abl as shown by the strong increase in autophosphorylation or phosphorylation of Y412 of Abl (Figure 3A, upper four panels). Furthermore, Mig6 was phosphorylated upon Abl overexpression indicating that it is also a substrate of c-Abl (Figure 3A, middle three panels). We then asked whether the Abl/Mig6 interaction could be detected at the endogenous level in pMECs and found that Mig6 coimmunoprecipitated with c-Abl both in the presence and absence of EGF, in a manner that could be partially prevented by nilotinib (Figure 3B). In addition, we incubated bacterially produced Mig6 with immunopurified Abl, followed by immunoprecipitation of c-Abl and western analysis for binding of Mig6 and phosphorylation of c-Abl (Figure S2H). Although Abl bound effectively to recombinant Mig6, only a modest 2.2-fold activation of Abl in the presence of Mig6 was observed, raising the possibility that additional proteins may be required for full Mig6-induced activation (Figure S3H). Taken together, these results reveal that Mig6 binds directly to and activates c-Abl.

We next investigated the ability of EGFR to coimmunoprecipitate with Mig6 and found that the two proteins bind in the presence but not absence of EGF (Figure 3C). Ectopically expressed Mig6 or mutant Mig6 deficient for Grb2 binding (PP319/20AA) coimmunoprecipitated with EGFR and inhibited EGFR autophosphorylation, as shown by western analysis (Figure 2D). On the contrary, Mig6 lacking the short EGFR interaction motif homologous to Ack1, the AH1 domain (Frosi et al., 2010; Zhang et al., 2007a), failed to bind to and inhibit EGFR (Figure 2D). These results are consistent with the model proposed by Kuriyan and coworkers (Zhang et al., 2007a) that Mig6 binds to the activating EGFR kinase domain interface, thereby attenuating autoactivation, and that Mig6 dissociates from EGFR upon receptor inactivation.

To address whether Mig6-induced activation of Abl could be regulated by EGFR activity, we ectopically expressed Abl with Mig6, in the presence or absence of active EGFR and analyzed Abl activation. Overexpression of active EGFR effectively prevented Mig6 binding to and activation of Abl (Figure 3E). This is consistent with a switch-like mechanism, whereby Mig6 either interacts with and attenuates EGFR activity or, upon inactivation/absence of EGFR, activates c-Abl.

To further characterize the molecular interaction between Mig6 and c-Abl, we ectopically expressed a series of deletion mutants of c-Abl and assessed their ability to coimmunoprecipitate with Mig6. Contrary to previously described Abl interactions, Mig6 does not bind to the SH3, SH2 domain or the last exon region (outlined in Figure S3A), but interacts with the inactive (kinase-dead) c-Abl kinase domain and less efficiently with the wild-type kinase domain (Figure S3B). In agreement, Mig6 was still able to interact with c-Abl point mutants that disrupt phosphotyrosine- or PxxP-ligand binding to the SH2 (R171L) or the SH3 (W118A) domains, respectively (Figure S3C).

### Mig6 Activates Abl by Interacting with the Kinase Domain via Its Conserved ErbB Binding Region

**Figure 4:**
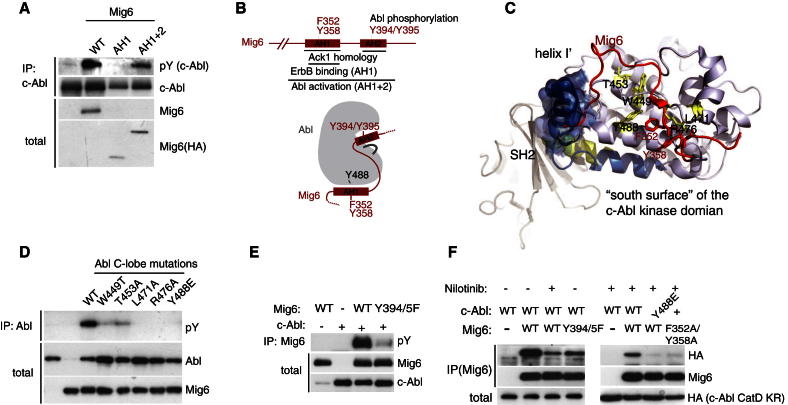
Mig6 Binds the Abl Kinase Domain via the Conserved EGFR Binding Region (A) Coimmunopurification of ectopically expressed c-Abl and full length (WT) Mig6 or fragments of HA-tagged Mig6 constituting either the minimal ErbB interaction aa 335–365 (Ack homology region 1, AH1) or the complete Ack homology region aa 314–412 (AH1+2). (B) Outline of the minimal region of Mig6 required for Abl activation with point mutations generated. (C) Molecular modeling of the predicted c-Abl/Mig6 interface based on the structural similarity with EGFR bound to Mig6 (EGFR/Mig6: PDB 2RF9 and c-Abl: PDB 1OPK). View of the c-Abl kinase domain (light blue) and Mig6 (red) with residues F352 and Y358 (highlighted red) known to contribute to the Mig6/EGFR interface. The regulatory helix I′ of c-Abl is shown in its inactive closed conformation (blue, PDB 1OPK) or active open conformation (yellow, PDB 1OPJ). Note the apparent clash between Mig6 and the inactive but not active I′ helix conformation. c-Abl residues predicted to participate in the interaction with Mig6 that were mutated in d are highlighted yellow. (D) Coimmunopurification of ectopically expressed Mig6 with mutant forms of c-Abl with indicated point mutations, implicating the predicted C-lobe interface is in the activation of Abl by Mig6. (E) Identification of the Abl phosphorylation sites on Mig6. Immunopurification of ectopically expressed wild-type or Y394/395F mutants of Mig6 and constitutively active c-Abl-PP. (F) Coimmunopurification of ectopically expressed HA-tagged inactive c-Abl kinase domains with indicated C-lobe mutation with WT or mutated forms of Mig6 in the presence or absence of nilotinib (2 μM). See also Figure S3.

Given the unique ability of Mig6 to interact directly with and activate the c-Abl kinase domain, we asked whether the interaction site is similar to that of Mig6 on the EGFR kinase domain, which was revealed by the crystal structure of the EGFR kinase domain bound to a fragment of Mig6 (Jura et al., 2009; Zhang et al., 2007a). We found that ectopic expression of a short extended peptide containing the EGFR-binding-AH1 domain (AH1+2), although not AH1 alone, was sufficient to trigger c-Abl activation (Figures 4A and 4B). This points toward a similar interaction interface for Mig6/c-Abl and Mig6/EGFR. Superimposition of structures of the EGFR and c-Abl kinase domains revealed a strong conservation in the Mig6 binding region of EGFR and the corresponding region of c-Abl. In total, six of eight critical Mig6-contact residues are conserved between Abl and EGFR and display a similar spatial orientation (Figures S3D and S3E). To address whether Mig6 interacts with c-Abl as predicted based on the analogy with Mig6 binding to the EGFR, we generated point mutants of predicted contact residues on the C-lobe of the Abl kinase domain (schematically outlined in Figure 4C) and assessed their ability to interact with and become activated by Mig6 (Figure 4D). All these point mutations prevented Mig6-mediated activation of Abl, indicating the involvement of the Abl kinase domain C-lobe in the functional interaction with Mig6 (Figure 4D). These mutations did not, however, prevent Mig6 from interacting with Abl implying the existence of another binding site.

Since Mig6 preferentially interacts with the inactive c-Abl kinase domain, rather than the active kinase that phosphorylates Mig6 (Figure S3B), we speculated that Mig6 additionally binds to the catalytic site of Abl, leading to Mig6 phosphorylation and dissociation from the active site. To identify the region of Mig6 that interacts with the Abl catalytic site, we first mapped the Abl phosphorylation sites on Mig6 to Y394/5 within the Ack1 homology region (outlined in Figure 2B). Mutation of these phosphorylation sites (Y394/5) to alanine eliminated Abl-induced phosphorylation of Mig6 (Figure 4E). The Y394/5F mutations partially abolished Mig6 binding to the inactive Abl kinase domain suggesting that Mig6 binds to the Abl catalytic site via the Ack1 homology domain (Figure 4F). As predicted, nilotinib, which binds the Abl catalytic site causing major misplacement of the activation loop also reduced the Mig6-Abl interaction (Figure 4F). Residual binding of Mig6 to the Abl catalytic site, that was observed in the presence of nilotinib, was abolished by mutations predicted to disrupt the C-lobe binding interface: Abl (Y488E) and Mig6 (F352A/Y358A) (Figure 4F). Collectively, these results show that the Ack1 homology region of Mig6 binds both to the catalytic site and the C-lobe of Abl. Interestingly, molecular modeling indicates that the autoinhibited c-Abl conformation is not compatible with Mig6 binding, as there are clashes with Mig6 and the bent I′ helix in the Abl kinase domain (labeled blue in Figure 4C).

### Src-Family Kinases Negatively Regulate Mig6-Mediated Abl Activation

**Figure 5:**
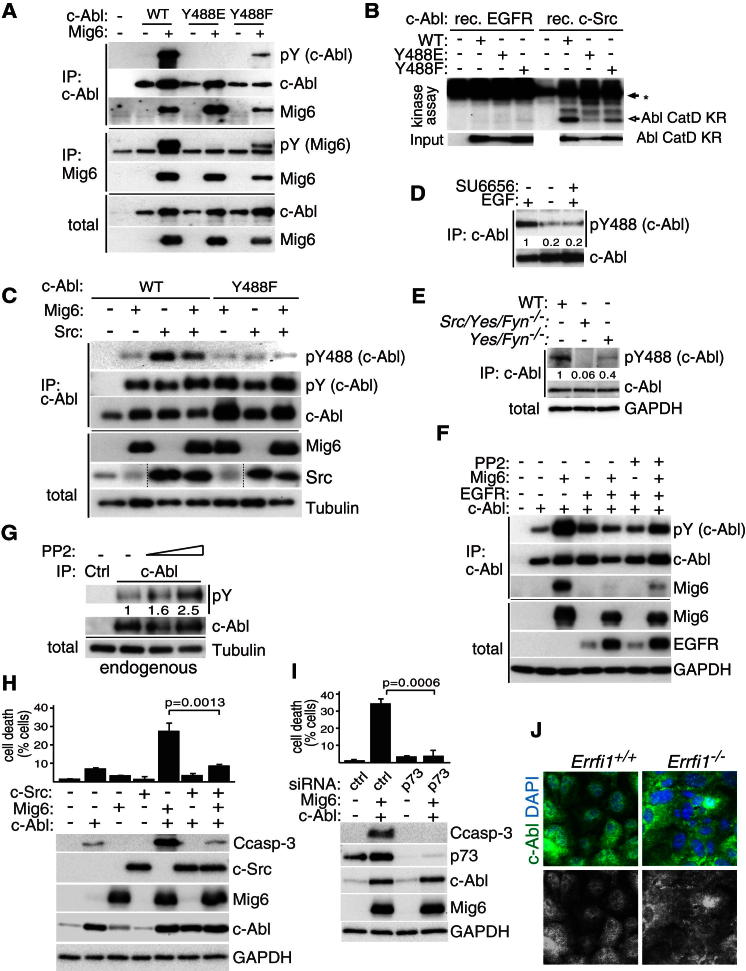
Phosphorylation of Abl on Y488 by Src-Kinases Regulates Mig6-Induced Activation of Abl (A) Coimmunopurification of ectopically expressed Mig6 with wild-type (WT), Y488E, or Y488F forms of c-Abl in HEK239T cells followed by western analysis as indicated. Note that the phosphomimic Y488E and partially the Y488F mutation disrupts the ability of Mig6 to activate c-Abl. (B) In vitro kinase assay with recombinant active c-Src or EGFR with wild-type (WT), Y488E or Y488F mutant forms of the inactive catalytic domain of c-Abl immunopurified from overexpressing HEK293T cells. (C) Immunoprecipitation of WT or Y488F mutant c-Abl coexpressed with Mig6 and active c-Src, followed by western analysis as indicated. (D) Western analysis of endogenous phosphorylation of Abl on Y488 in pMECs cultured in the presence or absence of EGF for 2 hr prior to cell harvest. Values denote relative intensities of p488 bands, normalized against total c-Abl levels. (E) Western analysis of endogenous phosphorylation of Abl on Y488 in control wild-type MEFs or MEFs lacking Src-family members. Values denote relative intensities of p488 bands, normalized against total c-Abl levels. (F) Immunopurification of c-Abl coexpressed with Mig6 and EGFR with or without 0.8 μM of the Src inhibitor PP2 and western analysis as indicated. Note the Src-dependent inhibition of Mig6-induced Abl activation by EGFR overexpression. (G) Western analysis of immunoprecipitated endogenous c-Abl for Y phosphorylation in pMEFs cultured in the presence of EGF, treated with 0.4 or 0.8 μM PP2 for 2 hr. Values denote relative intensities of pY bands, normalized against total immunoprecipitated c-Abl levels, as determined by densitometry. (H) Induction of cell death by Mig6/Abl but not Src/Abl. Ectopic expression of Abl with or without Mig6 or Src in pMEFs for 16 hr, followed by western analysis as indicated. Graph shows cell death determined as % of cells taking up the viability dye trypan blue (n = 3). (I) siRNA-mediated silencing of p73 followed by ectopic coexpression of Abl and Mig6 in pMEFs for 16 hr, and western analysis as indicated. Graph shows cell death determined as % of cells taking up the viability dye trypan blue (n = 3). (J) C-Abl immunofluorescence in WT or *Errfi1* KO pMECs cultured in absence of EGF for 2 hr. Note the reduced nuclear staining of c-Abl in the absence of Mig6. Images are representative of three independent experiments. Indicated p values were determined by two-tailed unpaired Student's t test, while the error bars represent SEM.

We noted that Abl-Y488, which is a known phosphorylation site with yet unknown function (Hantschel and Superti-Furga, 2004; Salomon et al., 2003), faces the predicted Mig6 interaction interface (highlighted in Figure 4C). This raises the possibility that phosphorylation of this site may regulate Mig6-induced activation. The Y488E mutation that mimics the phosphorylated tyrosine residue abolished the ability of Mig6 to activate c-Abl (Figure 5A), while the phenylalanine mutation (Y488F) led to decreased Mig6-mediated c-Abl activation (Figure 5A). We then asked whether either EGFR or c-Src, known to be recruited to and contribute to full activation of the ErbB receptors (Tice et al., 1999; Kim et al., 2005; Sato et al., 1995) could be responsible for phosphorylating c-Abl on Y488 and hence negatively regulate the activation of c-Abl by Mig6. Indeed, recombinant active c-Src but not EGFR was able to phosphorylate the c-Abl catalytic domain (Figure 5B). The Y488E/F mutations largely prevented Abl phosphorylation by Src indicating that it is a major Src phosphorylation site (Figure 5B). This was further confirmed using a phosphospecific antibody generated against pY488. Ectopically expressed Src, but not Abl itself activated by Mig6, phosphorylated Abl on Y488 (Figure 5C). Furthermore, we also found that endogenous c-Abl is phosphorylated on Y488 in the presence of EGF, while removal of EGF or inhibition of Src-family kinases with the SU6656 inhibitor lead to an 80% reduction in Y488 phosphorylation (Figure 5D). To confirm if c-Src and/or its closely related family members Yes and Fyn are responsible for phosphorylating endogenous c-Abl, we examined c-Abl-Y488 phosphorylation in mouse embryo fibroblasts (MEFs) from Src/Yes/Fyn triple or Yes/Fyn double knockout mice. Yes/Fyn^−/−^ MEFs shown a 60% reduction, while Src/Yes/Fyn^−/−^ MEFs fully abolish c-Abl-Y488 phosphorylation (Figure 5E). This indicates that endogenous Src, Yes, and Fyn contribute to phosphorylation of Abl-Y488. In addition, the Src family inhibitor PP2 partially restored the ability of Mig6 to activate c-Abl under conditions when EGFR was overexpressed (Figure 5F). At the endogenous level, we similarly found that Src inhibition by PP2 treatment lead to a 2.5-fold increase in c-Abl autophosphorylation (Figure 5G). These results suggest that c-Src kinases, prevents Mig6-dependent proapoptotic activation of c-Abl in the presence of EGF by phosphorylating Abl on Y488.

### Mig6/Abl but Not Src/Abl Triggers p73-Dependent Cell Death

We next addressed whether Abl activated by Mig6 is sufficient to trigger cell death by ectopic overexpression in primary MEFs. Coexpression of Mig6/Abl, but neither alone, nor coexpression of active Src/Abl, induced efficient cell death in *Errfi1*^*−/−*^ MEFs, as evident by immunoblotting for cleavage of caspase-3 or plasma membrane permeability (Figure 5H). In agreement with a role for Src-kinases in negatively regulating Mig6/Abl activity, overexpression of Src, lead to a 69% reduction in Mig6/Abl-induced cell death (Figure 5H).

To gain insight into the mechanism by which Mig6/Abl triggers cell death, we asked whether p73, which is an essential proapoptotic target of Abl in response to genotoxic stress, is also required for Mig6/Abl-induced death. Indeed, siRNA-mediated silencing of p73 reduced cell death induced by ectopic Mig6/Abl by 88% (Figure 5I). Nuclear accumulation of Abl has been reported to be important for its proapoptotic function in response to genotoxic stress, by allowing Abl to phosphorylate nuclear p73 (Agami et al., 1999; Chau et al., 2004; Gong et al., 1999). We therefore examined the cellular localization of Abl in wild-type and *Errfi1*^*−/−*^ pMECs by immunofluorescence. Abl localize in the cytoplasm and nucleus of wild-type pMECs, both in the absence (Figure 5J) or presence of EGF (data not shown). *Errfi1*^*−/−*^ cells exhibited a marked reduction in nuclear c-Abl as compared with wild-type cells (Figure 5J). This result raises the possibility that Mig6 is required for targeting Abl to the nucleus thereby allowing it to activate nuclear p73.

### Pharmacological Inhibition or RNAi Silencing of c-Abl Leads to Impaired Mammary Epithelial Cell Death and Lumen Formation

**Figure 6:**
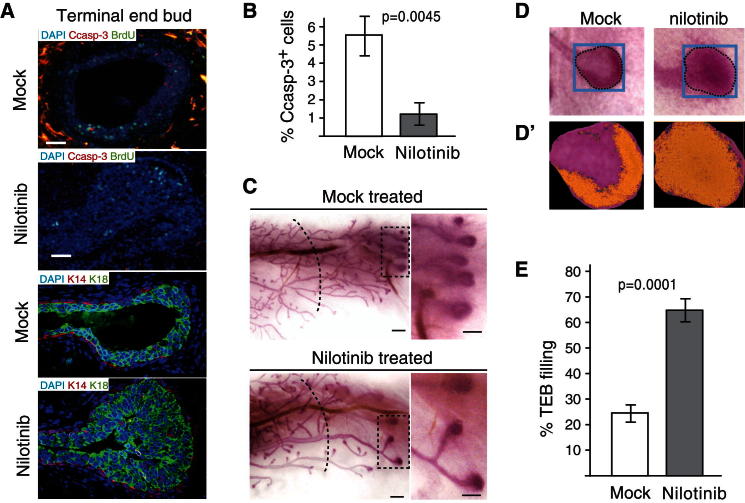
Abl Inhibition Impairs Luminal Cell Death and Cause TEB Filling (A) Immunostaining of TEBs for proliferating (BrdU-positive), cleaved caspase 3 (Ccasp-3) positive cells, or epithelial markers keratin 14 (K14) or keratin 18 (K18) of mice treated with nilotinib or vehicle (representative of three independent experiments). Scale bars = 20 μm. (B) Quantification of cleaved caspase 3 positive cells in TEBs of mock or nilotinib-injected mammary glands, n = 5 TEBs each sample group from three independent experiments. (C–D′) Whole-mount carmine staining of mammary glands from mock- or nilotinib-injected littermate mice. (C) The dotted line indicates the estimated position of the TEBs when treatment was initiated. Note filling of TEBs and reduced outgrowth of the secondary side branches upon nilotinib treatment. Scale bars = 500 μm. (D) The degree of filling of TEBs was quantified as the ratio of higher intensity areas of carmine staining over the total TEB area (as outlined, D′). (E) Quantification of degree of TEB filling in nilotinib-treated mice, n = 16 (nilotinib) and 12 (mock). In (B) and (E), data were analyzed by two-tailed unpaired Student's t test while error bars represent SEM.

To confirm whether c-Abl is required for cell death during mammary ductal morphogenesis, we treated mice with nilotinib daily for a week and analyzed the developing mammary glands. Longitudinal cross sections of TEBs were immunostained using antibodies against K14, K18, BrdU, and cleaved caspase 3, to determine the degree of luminal filling, cell death, proliferation, and differentiation (Figure 6A). Similar to the defects observed in the *Errfi1*^*−/−*^ mice, nilotinib treatment resulted in a 82% (p = 0.0045) reduction in the number of cleaved caspase 3 positive cells (Figures 6A and 6B) and a 2.8-fold (p = 0.0001) increase in luminal filling of TEBs (Figure 6E). The degree of filling was quantified by optical density analysis of carmine stained TEBs (Figures 6C–6E). Furthermore, nilotinib prevented outgrowth of the secondary branches but not the larger primary ducts, raising the possibility that Abl is additionally required for cell proliferation of secondary but not primary ducts (Figure 6C). Taken together these results show that nilotinib treatment phenocopies the impaired cell death and luminal filling observed in *Errfi1*^*−/−*^ TEBs.

**Figure 7:**
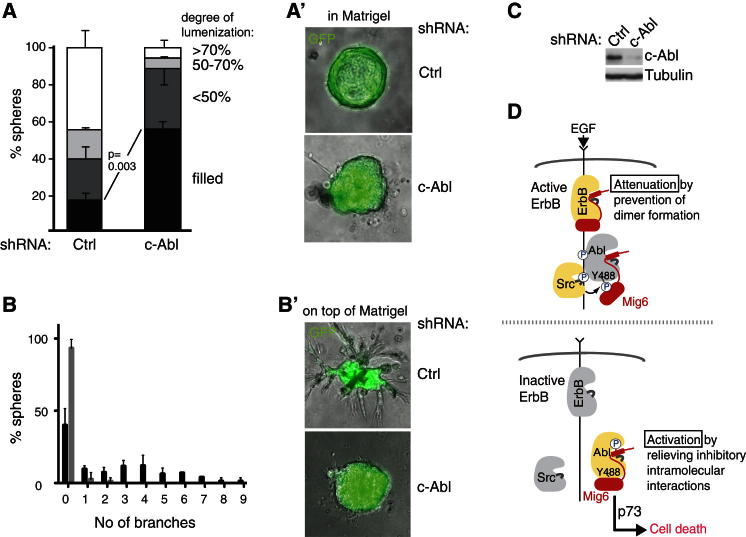
Silencing of c-Abl Leads to Impaired Mammary Lumen Formation and Branching Morphogenesis (A–C) pMECs were subjected to lentivirus-mediated shRNA silencing of c-Abl and cultured inside (A and A′) or on top of (B and B′) Matrigel. (A) The degree of luminal filling of individual acini was determined in a blind fashion from stacks of phase contrast and GFP fluorescence images (see A′ for representative acini). The proportion of spheres within the indicated subcategories are represented (n = 3 independent experiments with a total of 120 control and 118 Abl knockdown (k.d.) acini analyzed). (B) The number of branches of individual spheres cultured on top of Matrigel are plotted, n = 3 independent experiments with a total of 83 (ctrl) and 72 (Abl k.d.) acini analyzed. p = 0.006 for difference in mean number of branches per ctrl versus acini. (B′) Phase contrast/GFP images of representative acini. (C) Western analysis for indicated proteins of pMECs infected with the nontargeting or c-Abl-targeting shRNAs, confirming efficient knockdown. p values were determined by two-tailed unpaired Student's t test (A) or Mann-Whitney test (B), while error bars indicate SEM. (D) Model illustrating the molecular switch mechanism by which Mig6 senses ErbB receptor inactivation. In the presence of EGF the ErbB-binding region of Mig6 (red) binds to the active ErbB receptors interfering with kinase domain dimer formation (Zhang et al., 2007a). C-Src is recruited to and activated by active ErbB receptors and phosphorylates c-Abl on Y488, thereby preventing Mig6 from activating c-Abl to induce p73-dependent cell death. Upon ErbB receptor inactivation c-Abl and Mig6 dissociate from the receptor, leading to de-phosphorylation of c-Abl on Y488, which enable Mig6 to activate c-Abl to trigger p73-dependent cell death.

To confirm that the effect of nilotinib on mammary morphogenesis is due to inhibition of c-Abl we subjected pMECs to lentivirus-mediated shRNA silencing of Abl and cultured the cells in 3D Matrigel for 12 days and examined the formation of lumenized acini. This recapitulates aspects of in vivo mammary morphogenesis such as luminal clearance and ductal branching. The efficiency of lentiviral shRNA infection and knockdown was confirmed by green fluorescent protein (GFP) expression and immunoblotting for Abl (Figures 7A′, 7B′, and 7C). Silencing of Abl led to a significant (∼3-fold) increase in filled acini (Figures 7A and A′). Notably, the 14% of Abl knockdown acini that did form a lumen all exhibited a mosaic pattern of GFP expression (data not shown), suggesting that cells retaining Abl expression can support luminal clearance. In addition, pMEC acini cultured on top of, rather than inside, Matrigel extended branched structure in 46% of nontargeted control-infected acini, but only 9% of Abl knockdown acini (Figures 7B and B′). RNAi silencing of c-Abl in the 3D morphogenesis system can therefore recapitulate both the luminal filling and impaired branching phenotypes observed by nilotinib treatment or loss of Mig6 in mice. Notably, transplanting the Abl knockdown pMECs back into cleared fat pads failed to reconstitute the mammary gland, as opposed to the nontargeted control cells, suggesting that Abl has additional functions early during mammary morphogenesis (Figures S4A and S4B).

## Discussion

We have discovered an “inverse” mode of ErbB receptor signaling by which Mig6 monitors the activation state of the receptors to directly trigger apoptosis upon receptor inactivation. Our findings uncover a bimodal signaling function for Mig6, whereby in the presence of EGF it attenuates ErbB receptor dimerization while in the absence of EGF it directly binds to and activates c-Abl to induce cell death.

### Mig6 Is a Sensor of EGFR Activation Status that Creates Cellular Dependence on EGF

Lack of growth factors is widely thought to cause cell death passively due to loss of mitogenic and prosurvival canonical signaling cascades including the Ras/MAPK and PI3K/Akt pathways. However, this view is likely to be insufficient as many cell types are dependent on particular growth factors despite expressing multiple receptor tyrosine kinases, many of which activate the same canonical mitogenic signaling cascades. This has prompted the hypothetical definition of dependence receptors as those that promote a “positive” mitogenic signal upon ligand deprivation, but that actively induce a “negative” proapoptotic signal in the absence of ligand stimulation, such as Ret, Met, p75NTR, and EphA4 (Bredesen et al., 2004; Mehlen and Bredesen, 2004; Furne et al., 2009). One molecular mechanism underlying the negative receptor tyrosine kinase (RTK) signaling has in some cases been proposed to involve receptor cleavage by caspases that leads to further activation of caspases (Bredesen et al., 2004; Mehlen and Bredesen, 2004). However, the mechanism by which the caspases are activated by RTK inactivation in the first place and how the cleaved receptors propagate apoptosis remains unclear. Here, we show that the proapoptotic c-Abl kinase is rapidly activated upon ErbB receptor inactivation to trigger p73-dependent cell death. This involves a unique receptor-proximal sensory signaling mechanism by which Mig6 directly activates Abl, prior to the activation of caspase-3 and -9, to trigger apoptosis in the absence of ErbB ligand, thereby establishing specific cellular dependence on EGF.

### A Unique Mechanism of Kinase Activation

The adaptor protein Mig6 is known to specifically interact with members of the EGFR family of RTKs, in their active but not inactive state, to attenuate sustained kinase activity by disrupting asymmetric dimer formation. We show that Mig6 also interacts with the Abl kinase domain via the same interface as EGFR, but with opposite consequences, i.e., inhibition of EGFR activation but activation of Abl kinase activity. Notably, the mechanism by which Mig6 activates Abl is novel since all previously known substrates and/or activators of Abl bind either to the SH2 or SH3 domains or last exon region of Abl. The activation mechanism involves binding of Mig6 to the C-lobe of the Abl kinase domain. Similar to members of the Src family of kinases c-Abl is maintained in a tight autoinhibited conformation that is established by intimate intramolecular interactions involving the kinase domain and the SH2 and SH3 domains (Hantschel and Superti-Furga, 2004; Superti-Furga and Courtneidge, 1995). Molecular modeling indicates that the autoinhibited c-Abl conformation is not compatible with Mig6 binding, as there are clashes with Mig6 and the bent I′ helix in the Abl kinase domain binding. Our data are consistent with an induced fit mechanism of activation whereby Mig6 forces the regulatory helix (I′) to adopt the open conformation leading to a displacement of the SH3-SH2 domain unit and ensuing increase in tyrosine kinase activity and Abl autophosphorylation. Alternatively Mig6 binding could stabilize the open conformation, thereby shifting the balance from basal c-Abl activity toward autoactivation.

We found that Mig6 interacts with the active site of Abl in a manner that could be regulated by phosphorylation of Mig6 by Abl on Y394/5. Whether endogenous Mig6 binds EGFR and Abl in the same or separate complexes remains an open question. It is, however, plausible that Mig6 interacts simultaneously with inactive c-Abl via the catalytic site interface and active EGFR, while binding to the kinase domain C-lobe interfaces on EGFR or Abl are mutually exclusive.

We further provide evidence for the role of c-Src, and the closely related family members Yes and Fyn, in controlling the bimodal function of Mig6; in the presence of EGF c-Src kinases can phosphorylate Abl on Y488, which faces the predicted Mig6 interface, to prevent Mig6-dependent Abl activation. Intriguingly, previous studies have shown that c-Src can also contribute to c-Abl activation by phosphorylation on Y412 and Y245, two sites required for full Abl activation, to support growth factor-mediated mitogenic signaling (Dorey et al., 2001; Plattner et al., 1999). We find that ectopic Mig6/Abl, but not Src/Abl induces cell death in primary MEFs, raising the possibility that Abl regulated by RTK/Src show distinct “mitogenic” substrate preference as compared with “proapoptotic” substrates for Abl regulated by Mig6, hence providing a possible explanation for the apparent paradox that, depending on the cellular context, c-Abl can both support growth factor signaling and promote apoptosis. P73 may be such a specific Mig6/Abl target, given our observation that Mig6/Abl-induced cell death is prevented by RNAi silencing of p73. The mechanism by which Mig6/Abl could regulate p73 activity remains to be clarified; however, a possibility is that Mig6 is involved in targeting Abl to the nucleus where Abl has been described to phosphorylate and activate p73 in response to genotoxic stress (Agami et al., 1999; Chau et al., 2004; Gong et al., 1999). This is supported by our observation that c-Abl nuclear localization is impaired in *Errfi1*^*−/−*^ primary MECs.

### A Physiological Role for c-Abl/Mig6 in Promoting Cell Death during Epithelial Homeostasis

c-Abl is a multifunctional kinase that is regulated by integrins, cellular stresses and growth factors. Abl shuttles between different subcellular compartments including the cytoskeleton, cytoplasm, nucleus, and plasma membrane adding another level of complexity (Hantschel and Superti-Furga, 2004). Elucidating the biological functions of Abl has proved elusive primarily in part due to the embryonic or neonatal lethality of c-Abl knockout mice (Tybulewicz et al., 1991). Previous studies on cell lines suggest an important role for c-Abl in promoting cell death. Abl kinase activity is induced by various cellular stresses while loss of Abl function by RNAi, pharmacological inhibition of kinase activity and/or genetic deletion (using embryonic fibroblasts from knockout mice), leads to apoptotic defects in response to DNA damage, reactive oxygen species, ER stress and the proapototic cytokine TNFα (Agami et al., 1999; Chau et al., 2004; Gong et al., 1999; Ito et al., 2001; Noren et al., 2006; Sun et al., 2000). How Abl is activated by cellular stresses and how it triggers apoptosis remains largely unclear. Here, we provide evidence that Mig6-dependent Abl activity is required for cell death in epithelial cells during mammary ductal homeostasis in response to inactivation of EGFR. Pharmacological inhibition or RNAi-mediated knockdown of Abl leads to impaired ductal branching and epithelial cell death with subsequent luminal filling, either in vivo or in a 3D system of mammary morphogenesis. Notably, tissue-specific knockdown of Abl by transplantation of shRNA-targeted pMECs into cleared mammary fat pads prevented ductal outgrowth, suggesting that Abl has additional functions required for early mammary gland outgrowth.

The physiological relevance of ErbB receptor inactivation for the process of mammary epithelial cell death and lumen formation is supported by our observation that the luminal cell compartment that frequently undergoes apoptosis in the interior of TEBs shows reduced immunoreactivity against active phospho-ErbB2. Furthermore, previous studies have shown that constitutively active ErbB2 leads to impaired apoptosis and luminal filling in a 3D model of mammary lumen formation (Debnath et al., 2002; Muthuswamy et al., 2001). Importantly, enhanced mitogenic signaling alone induced by oncogenes (Cyclin D1 or HPV E7) is not sufficient to induce luminal filling (Debnath et al., 2002), implying a specific requirement for an ErbB2-derived mitogenic signal.

This is consistent with our finding that a receptor proximal sensory mechanism directly links ErbB receptor inactivation to cell death during epithelial homeostasis independent of canonical mitogenic signaling pathways.

Finally, we and others previously showed that Mig6 is an important tumor suppressor since knockout mice are highly susceptible to cancer formation, while Mig6 expression is frequently lost in various human cancers (Ferby et al., 2006; Zhang et al., 2007b). Here, we uncover that Mig6 can acts as a double-edged tumor suppressor that not only attenuates ErbB-signaling but also directly triggers cells death when ErbB receptors are inactive. This is of particular interest considering that acquisition of growth factor-independent survival is likely to be an early step of cellular transformation (Hanahan and Weinberg, 2011).

## Experimental Procedures

### Mouse Lines

*Errfi1*^*+/−*^ mice were maintained in a mixed Sv129/C57BL/6 background. While transplantation of lentivirus-infected pMECs were performed in BALB/c mice. All experiments with mice were performed with approval from the local ethics committee and the home office.

### pMEC Isolation

Mammary glands were harvested from 8- to 12-week-old *Errfi1*^*−/−*^ and littermate control mice. Lymph nodes were excised and the glands placed into Leibowitz L15 medium (Invitrogen) containing 10% FCS prior to dissociation on a McIIwain Tissue Chopper (Mickle). Tissue was digested for 7 hr at 37°C in medium containing 2 mg/ml collagenase (Roche) and 0.02 g/ml hyaluronidase. Erythrocytes were removed with red blood cell lysis buffer (Sigma). Fibroblasts were removed during a 1 hr incubation in DMEM containing 10% FCS at 37°C. Organoids were sequentially incubated in 5 mg/ml dispase II for 5 min, Joklik's MEM, 0.02% w/v EDTA for 10 min, and TrypLE (Invitrogen) at 37°C until a single cell suspension was achieved. 0.1 mg/ml DNase I (Invitrogen) in L15 containing 10% FCS was added to single cell suspensions prior to filtering with a 40 μm cell strainer.

### Cell Culture and Transfection

pMECs were cultured in mammary epithelial cell basal medium (Promocell), 10 ng/ml EGF, 5 μg/ml insulin, 0.5 μg/ml hydrocortisone, 10 ng/ml Cholera Toxin, and 2% FCS on tissue culture plates precoated in 1% collagen in 0.02 M acetic acid overnight at 4°C. HEK293T and MEFs cells were cultured in DMEM supplemented with 10% FBS (PAA Laboratories) and transfected using the CalPhos Transfection kit (Clontech) or Jetprime (Polyplus), respectively.

### Mammary Gland Analysis

Mammary glands were fixed overnight at 4°C in 60% w/v methanol, 30% w/v chloroform, 10% acetic acid. Fixed tissue was stained in carmine aluminum 0.2% w/v carmine red, 0.5% w/v aluminum potassium sulfate for whole-mount imaging followed by routine procedures for paraffin embedding, sectioning, and staining with hematoxylin and eosin.

### Lentivirus Production and Transduction of pMECs

Standard procedures for lentivirus production and titration were used. Viral supernatants were concentrated by ultracentrifugation and pMECs were infected for 48 hr prior to in vitro experiments, or overnight on poly-Hema-coated tissue-culture dishes for cleared fat pad transplantations or 3D Matrigel cultures.

### 3D Matrigel Cultures

Lentivirus-infected pMECs (prepared as described above) were plated inside or on top of Matrigel (BD Biosciences), and cultured in complete mammary epithelial cell basal medium for 12 days prior to analysis. Notably we only observe branching of wild-type pMEC acini when grown on standard Matrigel but not growth-factor reduced Matrigel.

### Cleared Mammary Fat Pad Transplantation

Wild-type and *Errfi1*^*−/−*^ pMECs isolated from 12-week-old Sv129/C57BL6 virgin female mice were resuspended in L15 medium (Invitrogen) to a concentration of 50,000 cells in 10 μl L15 medium, followed by injection into cleared mammary fat pads of 21-day-old Sv129/C57BL6 mice. For transplantation of Abl shRNA-infected pMECs, cells were isolated from 8-week-old BALB/c mice, subjected to lentiviral transfection as described above and injected into the fourth mammary fat pad of 21-day-old BALB/c mice after removal of endogenous mammary tissue.

### Immunofluorescence

Sections (6 μm) were deparaffinized and boiled in 10 mM sodium citrate buffer and blocked in 5% goat serum and M.O.M. Ig blocking reagent (Invitrogen) before incubation with primary and fluorofor-conjugated secondary antibodies (listed in Supplemental Experimental Procedures). After washing in 0.1% Tween-20 in TBS sections were counterstained with 1 μg/ml DAPI, mounted and images taken using the Leica TCS SP. Images were modified by introducing a threshold to eliminate background due to autofluorecence.

### Immunoblotting

Cells were either lysed directly in SDS-sample buffer or prepared for coimmunoprecipitation (Co-IP) and subjected to SDS-PAGE and western blotting. Co-IP procedure and list of antibodies used are found in the Supplemental Experimental Procedures.

### c-Abl Tyrosine Kinase Assay

pMEC cell lysates were subjected to immunoprecipitation as described above. Immunocomplexes were incubated at 30°C for 20 min in kinase reaction buffer containing 20 mM Tris-HCl (pH 7.5), 10 mM MgCl_2_, 10% v/v glycerol, 1 mM DTT, 1.5 μg/10 μl Abltide (Millipore) peptide substrate, and 5 μC/10 μl reaction ^32^P-γ-ATP.

### Trypan Blue Exclusion Assay

pMECs from *Errfi1*^*−/−*^ or wild-type 8- to 12-week-old mice were cultured to 70%–90% subconfluency prior to 8 hr EGF deprivation. Cells were treated with 0.2% trypan blue (Invitrogen) for 3 min followed by determination of the ratio of blue cells over total number of cells by transmission microscopy.

### EGF Deprivation Assay

pMECs were grown for 3–7 days to 70%–90% subconfluency prior to substitution to either EGF-supplemented or EGF-deficient complete media and cultured for indicated times in the absence or presence of the inhibitors; 1 μM gefitinib, 1 μM lapatinib, and 1 μM nilotinib (LC Laboratories) or 1 μM AG825, 1 μM c-Met inhibitor II, or 10 μM SU6656 (Merck).

### In Vivo Nilotinib Treatment and BrdU Incorporation

*Errfi1*^*−/−*^ and littermate control mice were injected intraperitoneally with 75 μg/g nilotinib 1:1 v/v in polyethylene glycol-200 daily for 7 days and with 0.25 mg/g BrdU in PBS 2 hr prior to sacrifice.

### Anoikis and DNA Damage Assays

pMECs were resuspended in methyl cellulose and cultured on culture dishes precoated with 20 mg/ml polyhema. pMECs were cultured in mammary epithelial cell medium containing 1 μg/ml adriamycin for 24 hr before harvesting for protein.

## Supplemental Information

Document S1. Figures S1–S4 and Supplemental Experimental Procedures
