# Supplementary material for: Mig6 Is a Sensor of EGF Receptor Inactivation that Directly Activates c-Abl to Induce Apoptosis during Epithelial Homeostasis
Source: Dev Cell. 2012 Sep 11;23(3):547–59. doi: 10.1016/j.devcel.2012.08.001 (PMC3657149; doi:10.1016/j.devcel.2012.08.001)
Supplement: Document S1. Figures S1–S4 and Supplemental Experimental Procedures [file mmc1.pdf]

Developmental Cell, Volume *23*

Supplemental Information

Mig6 Is a Sensor of EGF Receptor Inactivation that Directly Activates c-Abl to Induce Apoptosis during Epithelial Homeostasis

Sarah Hopkins, Emma Linderoth, Oliver Hantschel, Paula Suarez-Henriques, Giulia Pilia, Howard Kendrick, Matthew J. Smalley, Giulio Superti-Furga, and Ingvar Ferby

Inventory of Supplemental Data

Figure S1

Relating to Figure 1, showing **i**) histological characterization of the mammary ductal morphogenesis defects in the *Errfi1*$^{-/-}$ mice and **ii**) normal proliferation rate of *Errfi1*$^{-/-}$ pMECs cultured in vitro.

Figure S2

Relating to Figure 2, showing **i**) normal apoptotic response of *Errfi1*$^{-/-}$ pMECs upon cell detachment and DNA damage induced by Adriamycin, **ii**) that *Errfi1*$^{-/-}$ MECs express wild-type levels of markers for mammary epithelial differentiation an display normal cellular morphology and **iii**) that EGF-independent survival displayed by *Errfi1*$^{-/-}$ cells is not due to a gain in basal EGFR signalling or canonical mitogenic signalling and finally that **iv**) the binding of Mig6 and Abl is direct.

Figure S3

Relating to Figure 4. **i**) Maps the region of Abl that binds Mig6 and **ii**) models the structural similarity of the predicted Abl-Mig6 contact residues based on the analogy with the EGFR-Mig6 structure.

Figure S4

Relating to Figure 7, showing that RNAi-mediated knockdown of c-Abl in vivo, prevents mammary outgrowth.

SUPPLEMENTAL FIGURES

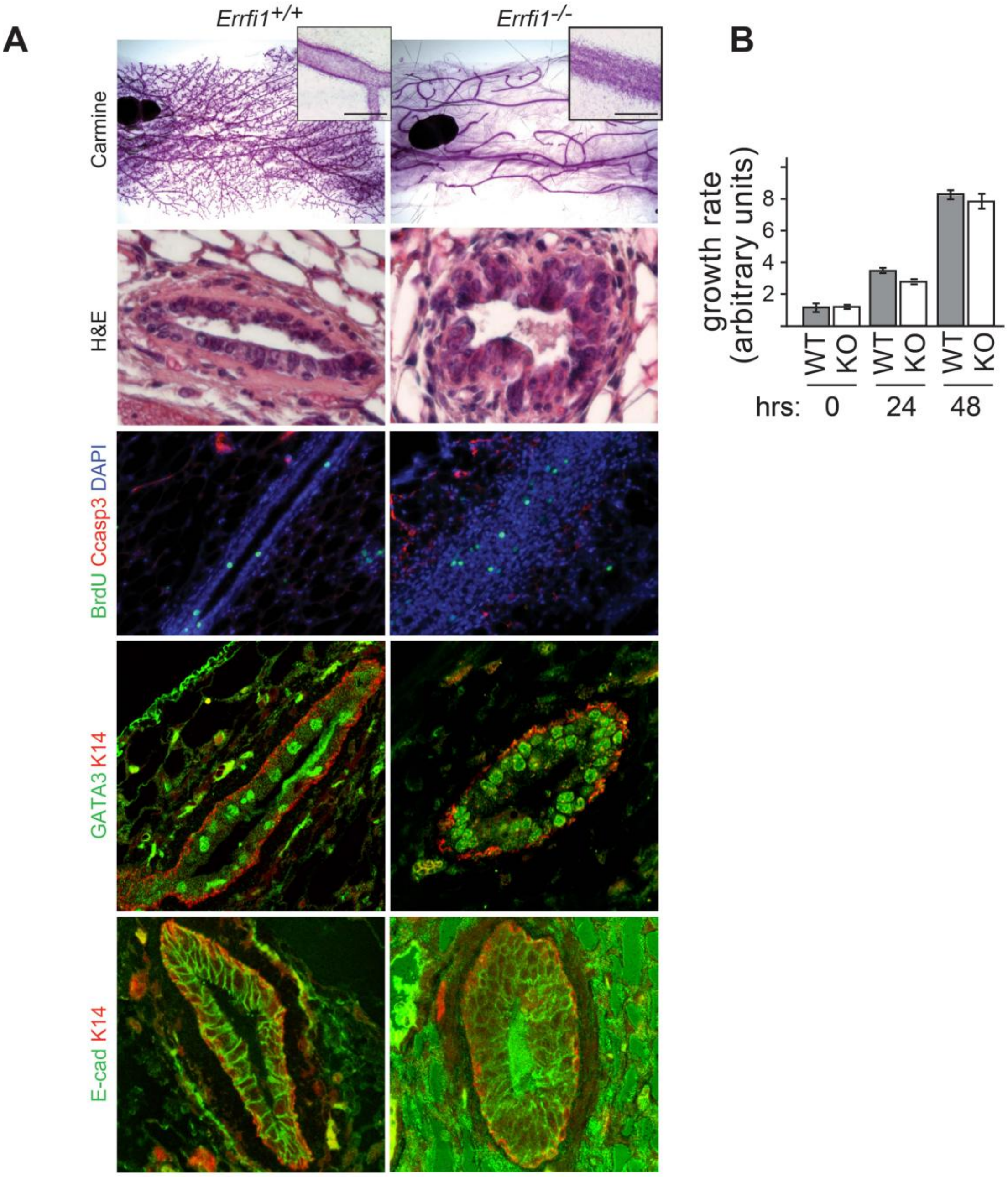

Figure S1 - Hopkins

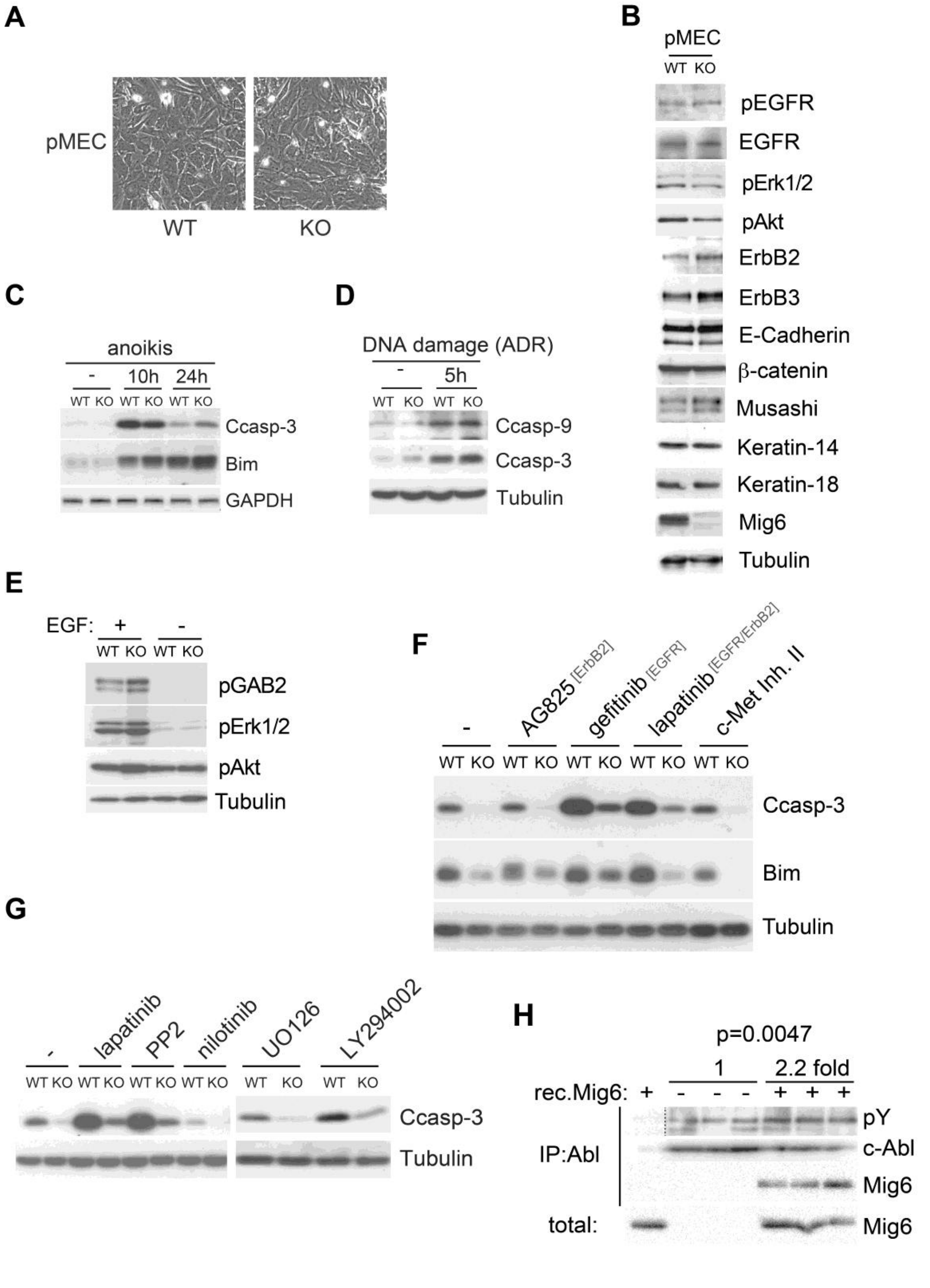

Figure S2 - Hopkins

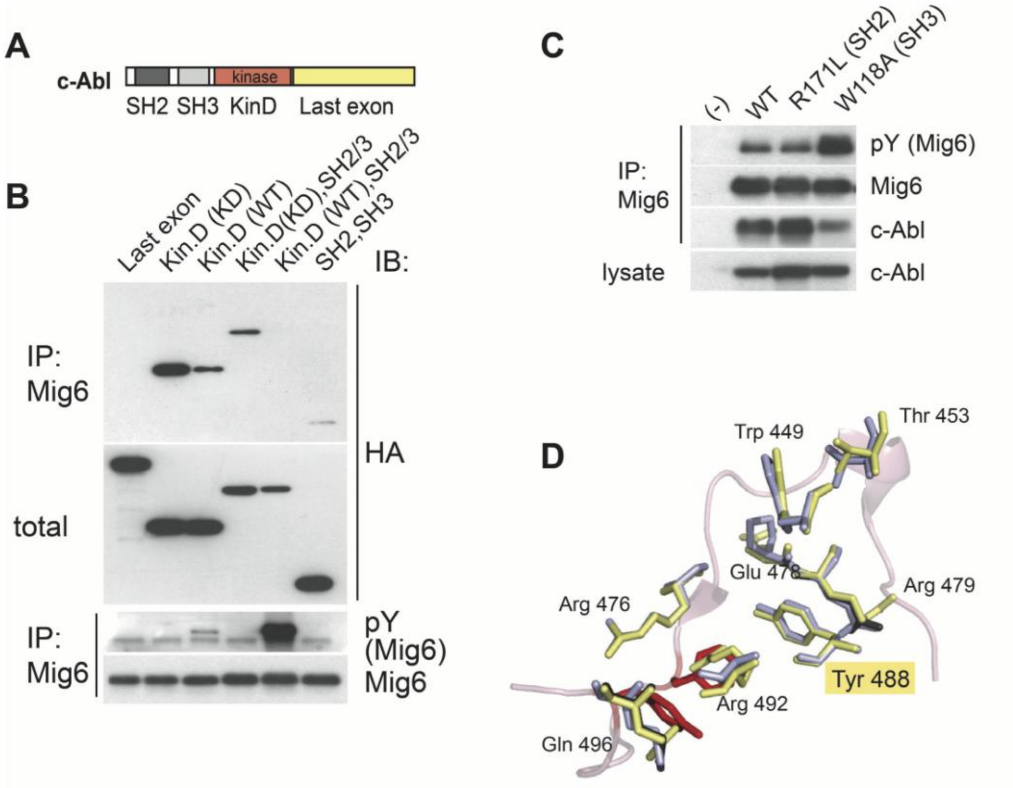

E

```
EGFR   904  VWELMTFGSKPYDGIPASEISSILEKGERLPQPPICTIDVYMIMVKCWM  952
             +WE+ T+G  PY GI  S++  +LEK  R+ +P  C   VY +M  CW+
c-Abl  448  LWEIATYGMSPYPGIDLSQVYELLEKDYRMERPEGCPEKVYELMRACWQ  496
```

Figure S3 - Hopkins

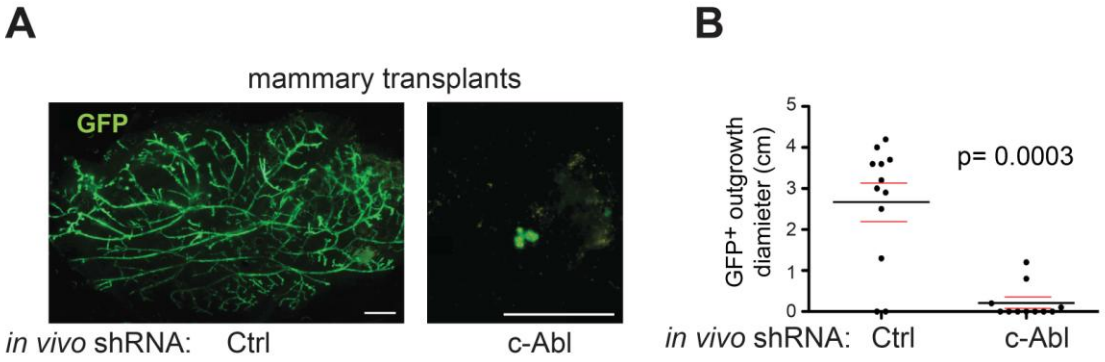

Figure S4 - Hopkins

FIGURE LEGENDS

Figure S1 related to Figure 1. Deletion of *Errfi1* leads to impaired mammary ductal morphogenesis and luminal filling. (A) Mammary glands from 12-week old *Errfi1*$^{-/-}$ or littermate wild-type female mice subjected to whole mount carmine staining (upper panels). Cross sections and longitudinal ductal sections from 10-week old mice were subjected to hematoxylin and eosin (H&E) staining (middle panels) immunostaining against indicated proteins. The images are representative of n=3 (BrdU/Ccasp3 and GATA3/K14) or n=4 (E-cadherin/K14) or n>8 (Carmine and H&E) mice. (B) *Errfi1*$^{-/-}$ or wild-type pMECs were cultured for indicated times and subjected to MTT reduction assay. Absorbance was measured at 570nm and units set arbitrary (n=4 independent experiments from 2 different mice). Error bars represent s.e.m. and indicated P-values were determined by two-tailed unpaired Student *t*-test.

Figure S2 related to Figure 2. Deletion of *Errfi1* does not affect mammary epithelial cell anoikis or apoptosis induced by genotoxic stress. (A) Phase contrast imaging of WT or *Errfi1* KO pMECs cultured for 6 days to confluence. Note the

normal morphology of KO pMECs. (B) Western analysis of cell lysates from confluent *Errfi1*$^{-/-}$ (KO) or littermate wild-type (WT) pMECs against indicated proteins cultured in the presence of EGF. (C,D) *Errfi1*$^{-/-}$ or wild-type pMECs isolated from 7-10 weeks old mice were either (A) cultured on cell repellent, poly-Hema-coated, tissue culture dishes (anoikis) or (B) treated with 1µg/ml adriamycin (ADR) for indicated times and cell lysates subjected to immunoblotting as indicated (n=2 independent experiments). (E) pMECs from WT or KO mice were either cultured in the presence or absence of EGF for 6 hours and analysed by western against indication proteins. (F) KO or littermate WT pMECs were deprived of EGF for 9 hours in the absence (-) or presence of 1µM AG825, 1µM Gefitinib, 1µM Lapatinib, 1µM c-Met inhibitor II, prior to western analysis for indicated proteins (representative of 2 independent experiments). (G) KO or littermate WT pMECs were deprived of EGF for 9 hours in the absence (-) or presence of 1µM Lapatinib, 0.8µM PP2, 2 µM nilotinib, 100nM UO126 or 1 µM LY294002, prior to western analysis for indicated proteins (representative of 2 independent experiments). (H) c-Abl was overexpressed in HEK293T cells, immunopurified, incubated with bacterially produced recombinant Mig6 (Abcam) and subjected to a kinase assay as described under Experimental Procedures. Samples were subjected to western analysis as shown. The indicated values denote fold change in pY signal intensity normalized against total c-Abl levels as determined by densitometry. The P-value were determined by two-tailed unpaired Student *t*-test (n=3).

Figure S3 related to Figure 4. Mapping the Mig6 interaction site on c-Abl (A) Outline of the domain structure of c-Abl **(B)** Co-immunopurification of Mig6 with HA-tagged fragments of c-Abl ectopically co-expressed in HEK293T cells, followed

by western analysis as indicated. The c-Abl fragments are the C-terminal last exon region, the wild-type catalytic domain alone (Kin.D), a point mutant catalytically inactive form of the kinase domain (Kin.D (KD)), the N-terminal SH2 and SH3 domains alone (SH2, SH3) or the wild-type and kinase inactive catalytic domain including the SH2 and –3 domains. Note that Mig6 binds preferentially to the catalytically inactive kinase domain. (C) Co-immunopurification of Mig6 with wildtype or mutant forms of c-Abl that disrupt the SH2 (R171L) or SH3 domain (W118A) expressed in HEK293T cells. (D) High similarity between the Mig6 binding site of EGFR and c-Abl. Molecular modeling shows that the identified contact residues of EGFR (blue) involved in Mig6 (pink) binding and the corresponding residues of c-Abl (yellow, and denoted) are strikingly similar; 4 out of 8 contact residues are identical while 2 represent conservative changes (EGFR/Mig6: PDB 2RF9 and c-Abl: PDB 1OPK). The non-conserved changes are: V924 and P510 in EGFR are R492 and E478 in c-Abl respectively. (E) Alignment of human EGFR and c-Abl protein sequences highlighting the conserved contact residues (boxed).

Figure S4, related to Figure 7. In vivo silencing of c-Abl leads to impaired mammary outgrowth. (A,B) Cleared fat pad transplants of pMECs infected with lentivirus carriying c-Abl-targeting shRNA or non-targeting control shRNA. GFP marks infected cells. (A) Representative fluorescence image of whole GFP-positive outgrowths. (B) The maximum diameter of individual GFP-positive mammary outgrowths: Ctrl (n=12) or Abl knock-down (n=11). Individual glands are plotted and black bar indicates the mean while red bars represent s.e.m. The p-value was determined by two-tailed unpaired Student *t*-test. Scale bars=500μm.

SUPPLEMENTAL EXPERIMENTAL PROCEDURES

cDNA constructs. Mammalian expression constructs with wild-type, point mutant and HA-tagged truncation mutant c-Abl1b were previously described (Hantschel et al., 2003). Mig6 was cloned into pcDNA4.1/TO and point mutants of Mig6: the F352A/Y358A, Y394/5F, deletion aa328-361, PP319/20AA as well as the c-Abl1b Y488E/F, W449T, L471A and R476A were generated by site-directed mutagenesis using the QuikChange XL Site-Directed Mutagenesis Kit (Agilent) and mutations confirmed by DNA sequencing. The HA-tagged Mig6 deletion mutants (AH1+2: aa314-403 and AH1: aa335-365) were cloned by PCR into pcDNA4.1/TO.

shRNA and siRNA. shRNA sequences used to target c-Abl were Abl#1: 5' AAAAGCAAGAACCTGTACACTTTCTTTCGAGAAAGTGTACAGGTTCTTGC3' Abl#1 or 5'AAAAGCAACAAGTCCACTATCTACGTTCGCGTAGATAGTGGGCTTGTTGC3' (Abl#2). Sequence of siRNA oligo targeting p73 was: GAATGAAAGTACCACCAAATT (Applied Biosystems).

Antibodies. The following antibodies were used for immunofluorescence: Cleaved Caspase-3, phopho-Y877-ErbB2 (Cell Signaling), Mig6 (Sigma), E-cadherin (BD Transduction Laboratories), Cytokeratin 14 (Covance), Cytokeratin 18 (Novocastra) GATA-3 (Santa Cruz) and anti-BrdU conjugated Alexa 488. Secondary antibodies used were Cy3, Cy5 (Molecular Probes) or Alexa 488 or Alexa 594 (Jackson ImmunoResearch)-conjugated antibodies. The following antibodies were used for western blotting: a phosphospecific c-Abl (Tyr488) antibody generated against

KVpYELMRA (Eurogentech), Mig6 (Sigma), α-Tubulin, EGFR, Cleaved Caspase-3 (Asp175), Cleaved Caspase-9 (Asp353) and Phospho-c-Abl Tyr412 (Cell Signaling), Bim (Abcam, ab7888), c-Abl1 (Hantschel et al., 2003), c-Abl1 (8E9), Phospho-cAbl Tyr245 and Phospho-Tyr (4G10). Secondary HRP conjugated antibodies were from Jackson ImmunoResearch.

SiRNA transfection of MEFs. Immortalized primary *Errfi1*$^{-/-}$ MEFs were grown in DMEM containing 10%FBS to 50-60% confluence and transfected with a siRNA oligo targeting p73 using Jetprime (Polyplus).

MTT cell proliferation assay. Freshly isolated pMECs from *Errfi1*$^{-/-}$ or wild-type 8-week old mice were cultured were seeded at a density of 30000 cells/cm^2 in 24-well plates and cultured for indicated times. 3-(-dimethylthiazol-2-yl) 2,5-diphenyl-tetrazolium bromide (MTT, Sigma) was added to the cells for 2 hours at 37C prior to harvest of cells and measurement of absorbance at 570nm.

Flow cytometry. pMECs were resuspended in mammary epithelial cell medium. Endothelial (CD31$^+$) cells and leukocytes (CD45$^+$) were labelled with rat anti-mouse CD31 antibody (MEC 13.3; BD Biosciences), anti-rat IgG microbeads and monoclonal anti-mouse CD45 conjugated microbeads (30f11.1; Miltenyi Biotec) and removed via magnetic cell separation using a MACS column and separator (Miltenyi Biotec). Separation of myoepithelial and luminal pMECs was obtained by incubation with anti-CD29 FITC (HM beta 1-1; AbD serotec) and biotin conjugated anti-CD61 (2C9.G2; BD Biosciences) in combination with APC-streptavidin (BD Biosciences) 4°C. Dead cells were excluded using 7-AAD.

Co-Immunoprecipitation. pMECs were lysed in 50mM Tris-HCL (pH7.5), 150mM NaCl, 1% v/v NP-40 and cleared by centrifugation at 9500g for 15 minutes. Soluble proteins were incubated with anti-c-Abl antibody (Hantschel et al., 2003) or anti-Mig6 antibody overnight at 4°C followed by a 2 hour incubation in protein G conjugated sepharose beads at 4°C. Immunocomplexes were washed twice with lysis buffer, twice with lysis buffer excluding NaCl and subjected to SDS-PAGE and western analysis of interacting proteins. Transfected HEK293T cells were lysed in RIPA lysis buffer (50 mM Tris-Cl pH 7.4, 150 mM NaCl, 1% NP-40, 0.5% sodium deoxycholate, 0.1% SDS) and insoluble material removed by centrifugation for 15 min at 9500g. Cleared lysates were incubated with anti-c-Abl (Hantschel et al., 2003), anti-Mig6 or anti-HA antibodies overnight at 4°C followed by a 1 hour incubation in protein G conjugated sepharose beads at 4°C. Immunocomplexes were washed four times with RIPA buffer and subjected to SDS-PAGE and western analysis for interacting proteins.

SUPPLEMENTAL REFERENCE

Hantschel, O., Nagar, B., Guettler, S., Kretzschmar, J., Dorey, K., Kuriyan, J., and Superti-Furga, G. (2003). A myristoyl/phosphotyrosine switch regulates c-Abl. Cell *112*, 845-857.
